# Supplementary material for: A role for CIM6P/IGF2 receptor in memory consolidation and enhancement
Source: eLife. 2020 May 5;9:e54781. doi: 10.7554/eLife.54781 (PMC7200152; doi:10.7554/eLife.54781)
Supplement: Source data 1. [file elife-54781-data1.docx]

**Source Data 1**

| **Fig. 1b** | **Effect of training on *Igf2r* mRNA** | | | | | | | | | | | | | |
| --- | --- | --- | --- | --- | --- | --- | --- | --- | --- | --- | --- | --- | --- | --- |
|  | Un | | | | | Tr | | | | | | | | |
| *Igf2r* levels (%) | 100.0 ± 13.46  (n = 5 rats) | | | | | 123.7 ± 11.64  (n = 5 rats) | | | | | | | | |
| Two-tailed Student's t-test: t (8) = 1.332, P = 0.2195 | | | | | | | | | | | | | | |
| **Fig. 1c** | **Effect of training on CIM6P/IGF2R protein - total and synapto** | | | | | | | | | | | | | |
|  | Un Total | Tr Total | | | | | Un Synapto | | | | | | Tr Synapto | |
| CIM6P/IGF2R levels (%) | 100 ± 12.48 (n = 8 rats) | 112.38 ± 10.11 (n = 8 rats) | | | | | 54.92 ± 4.34  (n = 8 rats) | | | | | | 49.08 ± 5.27  (n = 8 rats) | |
| One-way ANOVA followed by Tukey's post-hoc test  F (3, 28) = 13.26, P < 0.0001 | | | | | | | | | | | | | | |
| **Fig. 1d** | **Effect of training on CIM6P/IGF2R protein - timecourse** | | | | | | | | | | | | | |
|  | Un | 30 min | | | 2 day | | | | | 1 week | | | | 2 week |
| CIM6P/IGF2R levels (%) | 100 ±  16.76  (n = 8 rats) | 109.40 ± 22.50  (n = 6 rats) | | | 111.77 ± 25.91  (n = 6 rats) | | | | | 93.50 ± 22.31  (n = 6 rats) | | | | 94.64 ± 19.69  (n = 6 rats) |
| One-way ANOVA followed by Tukey's post-hoc test  F (4, 27) = 0.1469, P = 0.9628 | | | | | | | | | | | | | | |
| **Fig. 1–figure supplement 2** | **Effect of training on Egr1 protein - timecourse** | | | | | | | | | | | | | |
|  | Un | 30 min | | | 2 day | | | | | 1 week | | | | 2 week |
| Egr1 levels (%) | 100 ±  10.10  (n = 8 rats) | 188.01 ± 19.12  (n = 6 rats) | | | 107.06 ± 14.14  (n = 6 rats) | | | | | 100.59 ± 11.04  (n = 6 rats) | | | | 106.45 ± 14.64  (n = 6 rats) |
| One-way ANOVA followed by Tukey's post-hoc test  F (4, 27) = 14.12, P < 0.0001 | | | | | | | | | | | | | | |
| **Fig. 2a** | **Effect of two injections on IA memory** | | | | | | | | | | | | | |
|  | Mean Latency (s) | | | | | | | | | | | | | |
|  | Tr | T1 | | | | | | T2 | | | | T3 | | |
| IgG  (n = 6 rats) | 17.18 ± 5.74 | 301.51 ± 33.39 | | | | | | 276.92 ± 46.734 | | | | 311.84 ± 41.04 | | |
| Anti-CIM6P/IGF2R  (n = 6 rats) | 9.74 ± 2.52 | 9.74 ± 2.52 | | | | | | 61.84 ± 9.33 | | | | 69.72 ± 9.40 | | |
| Two-way RM ANOVA followed by Sidak's post-hoc test  Treatment: F (1, 10) = 30.47, P = 0.0003  Time: F (3, 30) = 48.49, P < 0.0001  Treatment x Test interaction: F (3, 30) = 19.30, P < 0.0001 | | | | | | | | | | | | | | |
| **Fig. 2b** | **Effect of single injection on IA memory** | | | | | | | | | | | | | |
|  | Mean Latency (s) | | | | | | | | | | | | | |
|  | Tr | | | T1 | | | | | | | T2 | | | |
| IgG  (n = 7 rats) | 17.56 ± 4.38 | | | 253.90 ± 31.32 | | | | | | | 301.54 ± 32.01 | | | |
| Anti-CIM6P/IGF2R  (n = 7 rats) | 16.56 ± 3.43 | | | 237.50 ± 30.49 | | | | | | | 279.03 ± 36.00 | | | |
| Two-way RM ANOVA followed by Sidak's post-hoc test  Treatment: F (1, 12) = 0.2657, P = 0.6156  Time: F (2, 24) = 75.93, P < 0.0001  Treatment x Test interaction: F (2, 24) = 0.1085 , P = 0.8976 | | | | | | | | | | | | | | |
| **Fig. 2c** | **Effect of 8 hr single injection on IA memory** | | | | | | | | | | | | | |
|  | Mean Latency (s) | | | | | | | | | | | | | |
|  | Tr | | | T1 | | | | | | | T2 | | | |
| IgG  (n = 6 rats) | 12.22 ± 5.08 | | | 319.97 ± 25.93 | | | | | | | 396.62 ± 27.48 | | | |
| Anti-CIM6P/IGF2R  (n = 6 rats) | 16.74 ± 2.95 | | | 333.58 ± 23.59 | | | | | | | 419.29 ± 19.41 | | | |
| Two-way RM ANOVA followed by Sidak's post-hoc test  Treatment: F (1, 10) = 0.5648, P = 0.4697  Time: F (2, 20) = 244.5, P < 0.0001  Treatment x Test interaction: F (2, 20) = 0.1167 , P = 0.8905 | | | | | | | | | | | | | | |
| **Fig. 2d** | **Effect of high dose single injection on IA memory** | | | | | | | | | | | | | |
|  | Mean Latency (s) | | | | | | | | | | | | | |
|  | Tr | | | T1 | | | | | | | T2 | | | |
| IgG  (n = 7 rats) | 16.38 ± 3.50 | | | 235.99 ± 32.96 | | | | | | | 292.12 ± 47.28 | | | |
| Anti-CIM6P/IGF2R  (n = 7 rats) | 18.51 ± 6.48 | | | 187.70 ± 25.83 | | | | | | | 220.37 ± 44.19 | | | |
| Two-way RM ANOVA followed by Sidak's post-hoc test  Treatment: F (1, 12) = 1.737, P = 0.2121  Time: F (2, 24) = 38.77, P < 0.0001  Treatment x Test interaction: F (2, 24) = 0.8564, P = 0.4372 | | | | | | | | | | | | | | |
| **Fig. 2e** | **Effect of 8 hr high dose single injection on IA memory** | | | | | | | | | | | | | |
|  | Mean Latency (s) | | | | | | | | | | | | | |
|  | Tr | | | T1 | | | | | | | T2 | | | |
| IgG  (n = 6 rats) | 14.18 ± 4.27 | | | 339.62 ± 29.46 | | | | | | | 385.63 ± 31.12 | | | |
| Anti-CIM6P/IGF2R  (n = 6 rats) | 18.00 ± 3.15 | | | 334.86 ± 22.96 | | | | | | | 397.12 ± 27.09 | | | |
| Two-way RM ANOVA followed by Sidak's post-hoc test  Treatment: F (1, 10) = 0.01673, P = 0.8997  Time: F (2, 20) = 362.5, P < 0.0001  Treatment x Test interaction: F (2, 20) = 0.1454, P = 0.8656 | | | | | | | | | | | | | | |
| **Fig. 2f** | **Effect of injection 15' before training on IA memory** | | | | | | | | | | | | | |
|  | Mean Latency (s) | | | | | | | | | | | | | |
|  | Tr | T1 | | | | | | T2 | | | | T3 | | |
| IgG  (n = 8 rats) | 17.14 ± 3.17 | 228.43 ± 21.83 | | | | | | 249.46 ± 35.73 | | | | 311.50 ± 49.43 | | |
| Anti-CIM6P/IGF2R  (n = 7 rats) | 13.96 ± 4.08 | 51.42 ± 13.35 | | | | | | 30.92 ± 7.77 | | | | 29.03 ± 8.36 | | |
| Two-way RM ANOVA followed by Sidak's post-hoc test  Treatment: F (1, 13) = 36.18, P < 0.0001  Time: F (3, 39) = 29.43, P < 0.0001  Treatment x Test interaction: F (3, 39) = 22.23, P < 0.0001 | | | | | | | | | | | | | | |
| **Fig. 3a** | **Effect of injection 15' before training (0.9 mA) on IA memory** | | | | | | | | | | | | | |
|  | Mean Latency (s) | | | | | | | | | | | | | |
|  | Tr | T1 | | | | | | T2 | | | | T3 | | |
| IgG  (n = 6 rats) | 17.14 ± 3.17 | 228.43 ± 21.83 | | | | | | 249.46 ± 35.73 | | | | 311.50 ± 49.43 | | |
| Anti-CIM6P/IGF2R  (n = 6 rats) | 13.96 ± 4.08 | 51.42 ± 13.35 | | | | | | 30.92 ± 7.77 | | | | 29.03 ± 8.36 | | |
| Two-way RM ANOVA followed by Sidak's post-hoc test  Treatment: F (1, 10) = 970.8, P < 0.0001  Time: F (3, 30) = 303.2, P < 0.0001  Treatment x Test interaction: F (3, 30) = 277.7, P < 0.0001 | | | | | | | | | | | | | | |
| **Fig. 3b** | **Effect of injection 15' before training (0.9mA) on 5' test** | | | | | | | | | | | | | |
|  | Mean Latency (s) | | | | | | | | | | | | | |
|  | Tr | | | | | | | | T1 | | | | | |
| IgG  (n = 6 rats) | 16.75 ± 3.90 | | | | | | | | 531.93 ± 24.32 | | | | | |
| Anti-CIM6P/IGF2R  (n = 6 rats) | 20.18 ± 3.90 | | | | | | | | 543.47 ± 24.60 | | | | | |
| Two-way RM ANOVA followed by Sidak's post-hoc test  Treatment: F (1, 10) = 0.1534, P = 0.7035  Time: F (1, 10) = 1085, P < 0.0001  Treatment x Test interaction: F (1, 10) = 0.06625, P = 0.8021 | | | | | | | | | | | | | | |
| **Fig. 3c** | **Effect of injection 15' before training (0.9mA) on 1 hr test** | | | | | | | | | | | | | |
|  | Mean Latency (s) | | | | | | | | | | | | | |
|  | Tr | | | | | | | | T1 | | | | | |
| IgG  (n = 6 rats) | 28.57 ± 7.46 | | | | | | | | 554.04 ± 18.95 | | | | | |
| Anti-CIM6P/IGF2R  (n = 6 rats) | 20.32 ± 4.98 | | | | | | | | 116.10 ± 11.17 | | | | | |
| Two-way RM ANOVA followed by Sidak's post-hoc test  Treatment: F (1, 10) = 375.0, P < 0.0001  Time: F (1, 10) = 645.5, P < 0.0001  Treatment x Test interaction: F (1, 10) = 308.8, P < 0.0001 | | | | | | | | | | | | | | |
| **Fig. 3d** | **Effect of injection 15' before training on Distance Travelled** | | | | | | | | | | | | | |
|  | IgG | | | | | Anti-CIM6P/IGF2R | | | | | | | | |
| Distance (cm) | 4069 ± 257.3  (n = 8 rats) | | | | | 3572 ± 185.0  (n = 8 rats) | | | | | | | | |
| Two-tailed Student's t-test: t (14) = 1.567 , P = 0.1394 | | | | | | | | | | | | | | |
| **Fig. 3e** | **Effect of injection 15' before training on Average Velocity** | | | | | | | | | | | | | |
|  | IgG | | | | | Anti-CIM6P/IGF2R | | | | | | | | |
| Velocity (cm/s) | 6.78 ± 0.43  (n = 8 rats) | | | | | 5.95 ± 0.31  (n = 8 rats) | | | | | | | | |
| Two-tailed Student's t-test: t (14) = 1.567, P = 0.1394 | | | | | | | | | | | | | | |
| **Fig. 3f** | **Effect of injection 15' before training on Time in Center** | | | | | | | | | | | | | |
|  | IgG | | | | | Anti-CIM6P/IGF2R | | | | | | | | |
| Cumulative time (s) | 9.40 ± 4.26  (n = 8 rats) | | | | | 6.61 ± 1.67  (n = 8 rats) | | | | | | | | |
| Two-tailed Student's t-test: t (14) = 0.6104, P = 0.5514 | | | | | | | | | | | | | | |
| **Fig. 3e** | **Effect of injection 15’ before 1 day test** | | | | | | | | | | | | | |
|  | Mean Latency (s) | | | | | | | | | | | | | |
|  | Tr | | | T1 | | | | | | | T2 | | | |
| IgG  (n = 6 rats) | 26.37 ± 6.30 | | | 550.08 ± 18.42 | | | | | | | 654.33 ± 19.42 | | | |
| Anti-CIM6P/IGF2R  (n = 6 rats) | 15.16 ± 2.95 | | | 538.93 ± 16.52 | | | | | | | 628.28 ± 14.93 | | | |
| Two-way RM ANOVA followed by Sidak's post-hoc test  Treatment: F (1, 10) = 1.570, P = 0.2388  Time: F (2, 20) = 1168, P < 0.0001  Treatment x Test interaction: F (2, 20) = 0.1932, P = 0.8259 | | | | | | | | | | | | | | |
| **Fig. 4b** | **Effect of injection Cre-injection on Distance Travelled** | | | | | | | | | | | | | |
|  | GFP control | | | | | Cre | | | | | | | | |
| Distance (cm) | 1878 ± 61.84  (n = 8 mice) | | | | | 2059 ± 233.8  (n = 8 mice) | | | | | | | | |
| Two-tailed Student's t-test: t (14) = 0.7496, P = 0.4659 | | | | | | | | | | | | | | |
|  | **Effect of injection Cre-injection on Average Velocity** | | | | | | | | | | | | | |
|  | GFP control | | | | | Cre | | | | | | | | |
| Velocity (cm/s) | 3.13 ± 0.10  (n = 8 mice) | | | | | 3.36 ± 0.37  (n = 8 mice) | | | | | | | | |
| Two-tailed Student's t-test: t (14) = 0.6138, P = 0.5492 | | | | | | | | | | | | | | |
|  | **Effect of Cre-injection on Time in Center** | | | | | | | | | | | | | |
|  | GFP control | | | | | Cre | | | | | | | | |
| Cumulative time (s) | 271.1 ± 33.00  (n = 8 mice) | | | | | 231.0 ± 34.81  (n = 8 mice) | | | | | | | | |
| Two-tailed Student's t-test: t (14) = 0.8346, P = 0.4180 | | | | | | | | | | | | | | |
| **Fig. 4c** | **Effect of Cre-injection on Novel Object Location** | | | | | | | | | | | | | |
|  | Preference (%) | | | | | | | | | | | | | |
|  | Tr | | | | | | | | T1 | | | | | |
| GFP control  (n = 8 mice) | 50.24 ± 1.11 | | | | | | | | 66.56 ± 2.17 | | | | | |
| Cre  (n = 8 mice) | 50.16 ± 1.36 | | | | | | | | 58.13 ± 1.34 | | | | | |
| Two-way RM ANOVA followed by Sidak's post-hoc test  Treatment: F (1, 14) = 61.24, P < 0.0001  Time: F (1, 14) = 7.582, P = 0.0155  Treatment x Test interaction: F (1, 14) = 7.232, P = 0.0176 | | | | | | | | | | | | | | |
|  | **Effect of Cre-injection on Total Exploration** | | | | | | | | | | | | | |
|  | GFP control | | | | | Cre | | | | | | | | |
| Exploration time (s) | 15.62 ± 0.96  (n = 8 mice) | | | | | 14.14 ± 0.81  (n = 8 mice) | | | | | | | | |
| Two-tailed Student's t-test: t (14) = 1.184 , P = 0.2562 | | | | | | | | | | | | | | |
| **Fig. 4d** | **Effect of Cre-injection on Context Test** | | | | | | | | | | | | | |
|  | Freezing (%) | | | | | | | | | | | | | |
|  | Tr | | | T1 | | | | | | | T2 | | | |
| GFP control  (n = 8 mice) | 5.21 ± 1.52 | | | 48.61 ± 2.73 | | | | | | | 52.78 ± 2.57 | | | |
| Cre  (n = 8 mice) | 3.13 ± 1.52 | | | 25 ± 3.15 | | | | | | | 23.61 ± 3.11 | | | |
| Two-way RM ANOVA followed by Sidak's post-hoc test  Treatment: F (1, 14) = 67.39, P < 0.0001  Time: F (1, 14) = 0.2979, P = 0.5938  Treatment x Test interaction: F (1, 14) = 1.191, P = 0.2935 | | | | | | | | | | | | | | |
| **Fig. 4e** | **Effect of Cre-injection on Tone test** | | | | | | | | | | | | | |
|  | Freezing (%) | | | | | | | | | | | | | |
|  | 24 hr Pre-tone | | 24 hr Tone | | | | | 1 week Pre-tone | | | | 1 week Tone | | |
| GFP control  (n = 8 mice) | 4.17 ± 2.73 | | 66.67 ± 3.52 | | | | | 6.25 ± 3.05 | | | | 65.62 ± 2.46 | | |
| Cre  (n = 8 mice) | 4.17 ± 2.73 | | 64.58 ± 3.05 | | | | | 8.33 ± 3.15 | | | | 63.54 ± 2.70 | | |
| Two-way RM ANOVA followed by Sidak's post-hoc test  Treatment: F (1, 14) = 0.03581, P = 0.8526  Time: F (3, 42) = 363.7, P < 0.0001  Treatment x Test interaction: F (3, 42) = 0.3074, P = 0.8199 | | | | | | | | | | | | | | |
| **Fig. 4f** | **Effect of Cre-injection on Context Test 1 min** | | | | | | | | | | | | | |
|  | Freezing (%) | | | | | | | | | | | | | |
|  | T1 | | | T2 | | | | | | | T3 | | | |
| GFP control  (n = 8 mice) | 43.75 ± 3.70 | | | 56.94 ± 2.52 | | | | | | | 56.25 ± 2.21 | | | |
| Cre  (n = 8 mice) | 43.75 ± 3.85 | | | 19.44 ± 3.79 | | | | | | | 14.58 ± 2.77 | | | |
| Two-way RM ANOVA followed by Sidak's post-hoc test  Treatment: F (1, 14) = 8.492e-013, P > 0.9999  Time: F (1, 14) = 228.5, P < 0.0001  Treatment x Test interaction: F (1, 14) = 7.833e-013, P > 0.9999 | | | | | | | | | | | | | | |
|  | **Effect of Cre-injection on Context Test 5 min** | | | | | | | | | | | | | |
|  | Freezing (%) | | | | | | | | | | | | | |
|  | Tr | | | | | | | | T1 | | | | | |
| GFP control  (n = 5 mice) | 1.67 ± 1.67 | | | | | | | | 52.22 ± 3.77 | | | | | |
| Cre  (n = 5 mice) | 1.67 ± 1.67 | | | | | | | | 21.11 ± 3.69 | | | | | |
| Two-way RM ANOVA followed by Sidak's post-hoc test  Treatment: F (1, 8) = 29.87, P = 0.0006  Time: F (1, 8) = 143.0, P < 0.0001  Treatment x Test interaction: F (1, 8) = 28.25, P = 0.0007 | | | | | | | | | | | | | | |
| **Fig. 4g** | **Effect of injection Cre-injection on CIM6P/IGF2R levels (WB)** | | | | | | | | | | | | | |
|  | GFP control | | | | | Cre | | | | | | | | |
| CIM6P/IGF2R levels (%) | 100.0 ± 9.26  (n = 8 mice) | | | | | 66.47 ± 5.97  (n = 7 mice) | | | | | | | | |
| Two-tailed Student's t-test: t (13) = 2.945 , P = 0.0114 | | | | | | | | | | | | | | |
|  |  |  | | |  | | | | |  | | | |  |
|  |  |  | | |  | | | | |  | | | |  |
| **Fig. 4h** | **Effect of injection Cre-injection on CIM6P/IGF2R levels (IHC) CA1** | | | | | | | | | | | | | |
|  | GFP control | | | | | Cre | | | | | | | | |
| CIM6P/IGF2R levels (%) | 100.0 ± 4.02  (n = 8 mice) | | | | | 62.07 ± 3.41  (n = 8 mice) | | | | | | | | |
| Two-tailed Student's t-test: t (14) = 7.191 , P < 0.0001 | | | | | | | | | | | | | | |
|  | **Effect of injection Cre-injection on CIM6P/IGF2R levels (IHC) DG** | | | | | | | | | | | | | |
|  | GFP control | | | | | Cre | | | | | | | | |
| CIM6P/IGF2R levels (%) | 100.0 ± 5.24  (n = 8 mice) | | | | | 58.76 ± 3.04  (n = 8 mice) | | | | | | | | |
| Two-tailed Student's t-test: t (14) = 6.811, P < 0.0001 | | | | | | | | | | | | | | |
| **Fig. 5a** | **Effect of training and anti-CIM6P/IGF2R on *Arc* mRNA** | | | | | | | | | | | | | |
|  | *Arc* levels (%) | | | | | | | | | | | | | |
|  | Un | | | | | | | | Tr | | | | | |
| IgG  (n = 5 rats) | 100 ± 24.26 | | | | | | | | 316.95 ± 55.57 | | | | | |
| Anti-CIM6P/IGF2R  (n = 5 rats) | 111.40 ± 27.11 | | | | | | | | 297.48 ± 37.44 | | | | | |
| Two-way RM ANOVA followed by Tukey's post-hoc test  Treatment: F (1, 16) = 0.01120, P = 0.9170  Training: F (1, 16) = 27.94, P < 0.0001  Treatment x Training interaction: F (1, 16) = 0.1639, P = 0.6910 | | | | | | | | | | | | | | |
|  | **Effect of training and anti-CIM6P/IGF2R on *Egr1* mRNA** | | | | | | | | | | | | | |
|  | *Egr1* levels (%) | | | | | | | | | | | | | |
|  | Un | | | | | | | | Tr | | | | | |
| IgG  (n = 5 rats) | 100 ± 8.52 | | | | | | | | 284.54 ± 42.48 | | | | | |
| Anti-CIM6P/IGF2R  (n = 5 rats) | 105.70 ± 9.81 | | | | | | | | 295.35 ± 30.55 | | | | | |
| Two-way RM ANOVA followed by Tukey's post-hoc test  Treatment: F (1, 16) = 0.09374, P = 0.7634  Training: F (1, 16) = 48.17, P < 0.0001  Treatment x Training interaction: F (1, 16) = 0.008965, P = 0.9257 | | | | | | | | | | | | | | |
|  | **Effect of training and anti-CIM6P/IGF2R on *c-Fos* mRNA** | | | | | | | | | | | | | |
|  | *c-Fos* levels (%) | | | | | | | | | | | | | |
|  | Un | | | | | | | | Tr | | | | | |
| IgG  (n = 5 rats) | 100 ± 18.44 | | | | | | | | 182.12 ± 24.19 | | | | | |
| Anti-CIM6P/IGF2R  (n = 5 rats) | 86.26 ± 11.13 | | | | | | | | 197.55 ± 15.71 | | | | | |
| Two-way RM ANOVA followed by Tukey's post-hoc test  Treatment: F (1, 16) = 0.002204, P = 0.9631  Training: F (1, 16) = 28.88, P < 0.0001  Treatment x Training interaction: F (1, 16) = 0.6564, P = 0.4297 | | | | | | | | | | | | | | |
| **Fig. 5b** | **Effect of training and anti-CIM6P/IGF2R on Arc protein intensity CA1** | | | | | | | | | | | | | |
|  | Arc levels (%) | | | | | | | | | | | | | |
|  | Un | | | | | | | | Tr | | | | | |
| IgG  (n = 4 rats) | 100 ± 4.05 | | | | | | | | 137.68 ± 10.02 | | | | | |
| Anti-CIM6P/IGF2R  (n = 4 rats) | 99.14 ± 3.98 | | | | | | | | 104.55 ± 4.17 | | | | | |
| Two-way RM ANOVA followed by Tukey's post-hoc test  Treatment: F (1, 12) = 7.700, P = 0.0168  Training: F (1, 12) = 12.37, P = 0.0042  Treatment x Training interaction: F (1, 12) = 6.942, P = 0.0218 | | | | | | | | | | | | | | |
|  | **Effect of training and anti-CIM6P/IGF2R on % Arc positive cells CA1** | | | | | | | | | | | | | |
|  | % Arc positive cells | | | | | | | | | | | | | |
|  | Un | | | | | | | | Tr | | | | | |
| IgG  (n = 4 rats) | 57.39 ± 1.99 | | | | | | | | 74.14 ± 2.35 | | | | | |
| Anti-CIM6P/IGF2R  (n = 4 rats) | 59.80 ± 1.38 | | | | | | | | 56.29 ± 1.30 | | | | | |
| Two-way RM ANOVA followed by Tukey's post-hoc test  Treatment: F (1, 12) = 18.23, P = 0.0011  Training: F (1, 12) = 13.38, P = 0.0033  Treatment x Training interaction: F (1, 12) = 31.39, P = 0.0001 | | | | | | | | | | | | | | |
|  | **Effect of training and anti-CIM6P/IGF2R on Arc protein intensity DG** | | | | | | | | | | | | | |
|  | Arc levels (%) | | | | | | | | | | | | | |
|  | Un | | | | | | | | Tr | | | | | |
| IgG  (n = 4 rats) | 100 ± 3.67 | | | | | | | | 165.17 ± 3.36 | | | | | |
| Anti-CIM6P/IGF2R  (n = 4 rats) | 109.39 ± 3.24 | | | | | | | | 110.79 ± 2.68 | | | | | |
| Two-way RM ANOVA followed by Tukey's post-hoc test  Treatment: F (1, 12) = 47.64, P < 0.0001  Training: F (1, 12) = 104.3, P < 0.0001  Treatment x Training interaction: F (1, 12) = 95.73, P < 0.0001 | | | | | | | | | | | | | | |
|  | **Effect of training and anti-CIM6P/IGF2R on % Arc positive cells DG** | | | | | | | | | | | | | |
|  | % Arc positive cells | | | | | | | | | | | | | |
|  | Un | | | | | | | | Tr | | | | | |
| IgG  (n = 4 rats) | 34.72 ± 1.98 | | | | | | | | 59.35 ± 2.71 | | | | | |
| Anti-CIM6P/IGF2R  (n = 4 rats) | 33.14 ± 2.53 | | | | | | | | 32.52 ± 1.87 | | | | | |
| Two-way RM ANOVA followed by Tukey's post-hoc test  Treatment: F (1, 12) = 38.15, P < 0.0001  Training: F (1, 12) = 27.24, P = 0.0002  Treatment x Training interaction: F (1, 12) = 30.14, P = 0.0001 | | | | | | | | | | | | | | |
| **Fig. 5c** | **Effect of training and anti-CIM6P/IGF2R on Egr1 protein intensity CA1** | | | | | | | | | | | | | |
|  | Egr1 levels (%) | | | | | | | | | | | | | |
|  | Un | | | | | | | | Tr | | | | | |
| IgG  (n = 4 rats) | 100 ± 4.29 | | | | | | | | 148.98 ± 6.09 | | | | | |
| Anti-CIM6P/IGF2R  (n = 4 rats) | 98.50 ± 5.46 | | | | | | | | 100.40 ± 2.26 | | | | | |
| Two-way RM ANOVA followed by Tukey's post-hoc test  Treatment: F (1, 12) = 27.74, P = 0.0002  Training: F (1, 12) = 28.62, P = 0.0002  Treatment x Training interaction: F (1, 12) = 24.52, P = 0.0003 | | | | | | | | | | | | | | |
|  | **Effect of training and anti-CIM6P/IGF2R on % Egr1 positive cells CA1** | | | | | | | | | | | | | |
|  | % Egr1 positive cells | | | | | | | | | | | | | |
|  | Un | | | | | | | | Tr | | | | | |
| IgG  (n = 4 rats) | 48.85 ± 3.73 | | | | | | | | 43.85 ± 8.15 | | | | | |
| Anti-CIM6P/IGF2R  (n = 4 rats) | 43.85 ± 8.15 | | | | | | | | 46.78 ± 3.42 | | | | | |
| Two-way RM ANOVA followed by Tukey's post-hoc test  Treatment: F (1, 12) = 0.02710, P = 0.8720  Training: F (1, 12) = 0.02710, P = 0.8720  Treatment x Training interaction: F (1, 12) = 0.3959, P = 0.5410 | | | | | | | | | | | | | | |
|  | **Effect of training and anti-CIM6P/IGF2R on Egr1 protein intensity DG** | | | | | | | | | | | | | |
|  | Egr1 levels (%) | | | | | | | | | | | | | |
|  | Un | | | | | | | | Tr | | | | | |
| IgG  (n = 4 rats) | 100 ± 3.87 | | | | | | | | 226.06 ± 27.14 | | | | | |
| Anti-CIM6P/IGF2R  (n = 4 rats) | 101.65 ± 3.72 | | | | | | | | 109.39 ± 9.09 | | | | | |
| Two-way RM ANOVA followed by Tukey's post-hoc test  Treatment: F (1, 12) = 15.60, P = 0.0019  Training: F (1, 12) = 21.11, P = 0.0006  Treatment x Training interaction: F (1, 12) = 16.50, P = 0.0016 | | | | | | | | | | | | | | |
|  | **Effect of training and anti-CIM6P/IGF2R on % Egr1 positive cells DG** | | | | | | | | | | | | | |
|  | % Egr1 positive cells | | | | | | | | | | | | | |
|  | Un | | | | | | | | Tr | | | | | |
| IgG  (n = 4 rats) | 12.42 ± 1.69 | | | | | | | | 76.07 ± 3.19 | | | | | |
| Anti-CIM6P/IGF2R  (n = 4 rats) | 9.94 ± 1.52 | | | | | | | | 7.62 ± 3.05 | | | | | |
| Two-way RM ANOVA followed by Tukey's post-hoc test  Treatment: F (1, 12) = 204.6, P < 0.0001  Training: F (1, 12) = 152.9, P < 0.0001  Treatment x Training interaction: F (1, 12) = 177.0, P < 0.0001 | | | | | | | | | | | | | | |
| **Fig. 5d** | **Effect of training and anti-CIM6P/IGF2R on c-Fos protein intensity CA1** | | | | | | | | | | | | | |
|  | c-Fos levels (%) | | | | | | | | | | | | | |
|  | Un | | | | | | | | Tr | | | | | |
| IgG  (n = 4 rats) | 100 ± 3.88 | | | | | | | | 152.55 ± 10.41 | | | | | |
| Anti-CIM6P/IGF2R  (n = 4 rats) | 99.26 ± 7.42 | | | | | | | | 103.15 ± 7.52 | | | | | |
| Two-way RM ANOVA followed by Tukey's post-hoc test  Treatment: F (1, 12) = 10.70, P = 0.0067  Training: F (1, 12) = 13.56, P = 0.0031  Treatment x Training interaction: F (1, 12) = 10.07, P = 0.0080 | | | | | | | | | | | | | | |
|  | **Effect of training and anti-CIM6P/IGF2R on % c-Fos positive cells CA1** | | | | | | | | | | | | | |
|  | % c-Fos positive cells | | | | | | | | | | | | | |
|  | Un | | | | | | | | Tr | | | | | |
| IgG  (n = 4 rats) | 64.88 ± 1.185 | | | | | | | | 70.545 ± 2.49 | | | | | |
| Anti-CIM6P/IGF2R  (n = 4 rats) | 66.85 ± 2.47 | | | | | | | | 67.47 ± 2.11 | | | | | |
| Two-way RM ANOVA followed by Tukey's post-hoc test  Treatment: F (1, 12) = 0.06677, P = 0.8005  Training: F (1, 12) = 2.174, P = 0.1661  Treatment x Training interaction: F (1, 12) = 1.404, P = 0.2590 | | | | | | | | | | | | | | |
|  | **Effect of training and anti-CIM6P/IGF2R on c-Fos protein intensity DG** | | | | | | | | | | | | | |
|  | c-Fos levels (%) | | | | | | | | | | | | | |
|  | Un | | | | | | | | Tr | | | | | |
| IgG  (n = 4 rats) | 100 ± 2.25 | | | | | | | | 98.66 ± 7.45 | | | | | |
| Anti-CIM6P/IGF2R  (n = 4 rats) | 184.44 ± 16.16 | | | | | | | | 102.64 ± 10.89 | | | | | |
| Two-way RM ANOVA followed by Tukey's post-hoc test  Treatment: F (1, 12) = 15.70, P = 0.0019  Training: F (1, 12) = 17.76, P = 0.0012  Treatment x Training interaction: F (1, 12) = 14.70, P = 0.0024 | | | | | | | | | | | | | | |
|  | **Effect of training and anti-CIM6P/IGF2R on % c-Fos positive cells DG** | | | | | | | | | | | | | |
|  | % c-Fos positive cells | | | | | | | | | | | | | |
|  | Un | | | | | | | | Tr | | | | | |
| IgG  (n = 4 rats) | 42.13 ± 1.11 | | | | | | | | 72.98 ± 4.40 | | | | | |
| Anti-CIM6P/IGF2R  (n = 4 rats) | 41.25 ± 1.55 | | | | | | | | 43.71 ± 2.02 | | | | | |
| Two-way RM ANOVA followed by Tukey's post-hoc test  Treatment: F (1, 12) = 31.94, P = 0.0001  Training: F (1, 12) = 39.08, P < 0.0001  Treatment x Training interaction: F (1, 12) = 29.37, P = 0.0002 | | | | | | | | | | | | | | |
| **Fig. 5- figure supplement 1a** | **Effect of training and anti-CIM6P/IGF2R on # of analyzed cells (Arc) CA1** | | | | | | | | | | | | | |
|  | # of DAPI-positive cells | | | | | | | | | | | | | |
|  | Un | | | | | | | | Tr | | | | | |
| IgG  (n = 4 rats) | 1482 ± 42 | | | | | | | | 1414 ± 42 | | | | | |
| Anti-CIM6P/IGF2R  (n = 4 rats) | 1522 ± 94.06 | | | | | | | | 1470 ± 42.50 | | | | | |
| Two-way RM ANOVA followed by Tukey's post-hoc test  Treatment: F (1, 12) = 1.015, P = 0.3335  Training: F (1, 12) = 0.6499, P = 0.4358  Treatment x Training interaction: F (1, 12) = 0.01805, P = 0.8953 | | | | | | | | | | | | | | |
|  | **Effect of training and anti-CIM6P/IGF2R on # of analyzed cells (Arc) DG** | | | | | | | | | | | | | |
|  | # of DAPI-positive cells | | | | | | | | | | | | | |
|  | Un | | | | | | | | Tr | | | | | |
| IgG  (n = 4 rats) | 2630 ± 93.55 | | | | | | | | 2624 ± 170.18 | | | | | |
| Anti-CIM6P/IGF2R  (n = 4 rats) | 2560 ± 86.29 | | | | | | | | 2440 ± 187.02 | | | | | |
| Two-way RM ANOVA followed by Tukey's post-hoc test  Treatment: F (1, 12) = 0.8051, P = 0.3872  Training: F (1, 12) = 0.1981, P = 0.6642  Treatment x Training interaction: F (1, 12) = 0.1622, P = 0.6942 | | | | | | | | | | | | | | |
| **Fig. 5- figure supplement 1b** | **Effect of training and anti-CIM6P/IGF2R on # of analyzed cells (Egr1) CA1** | | | | | | | | | | | | | |
|  | # of DAPI-positive cells | | | | | | | | | | | | | |
|  | Un | | | | | | | | Tr | | | | | |
| IgG  (n = 4 rats) | 1338 ± 52.40 | | | | | | | | 1386 ± 98.11 | | | | | |
| Anti-CIM6P/IGF2R  (n = 4 rats) | 1316 ± 25.46 | | | | | | | | 1288 ± 58.61 | | | | | |
| Two-way RM ANOVA followed by Tukey's post-hoc test  Treatment: F (1, 12) = 0.3511, P = 0.5645  Training: F (1, 12) = 0.02431, P = 0.8787  Treatment x Training interaction: F (1, 12) = 0.8752, P = 0.3680 | | | | | | | | | | | | | | |
|  | **Effect of training and anti-CIM6P/IGF2R on # of analyzed cells (Egr1) DG** | | | | | | | | | | | | | |
|  | # of DAPI-positive cells | | | | | | | | | | | | | |
|  | Un | | | | | | | | Tr | | | | | |
| IgG  (n = 4 rats) | 2472 ± 166.44 | | | | | | | | 2408 ± 69.21 | | | | | |
| Anti-CIM6P/IGF2R  (n = 4 rats) | 2380 ± 63.87 | | | | | | | | 2256 ± 142.02 | | | | | |
| Two-way RM ANOVA followed by Tukey's post-hoc test  Treatment: F (1, 12) = 0.6229, P = 0.4453  Training: F (1, 12) = 1.049, P = 0.3259  Treatment x Training interaction: F (1, 12) = 0.06345, P = 0.8054 | | | | | | | | | | | | | | |
| **Fig. 5- figure supplement 1c** | **Effect of training and anti-CIM6P/IGF2R on # of analyzed cells (c-Fos) CA1** | | | | | | | | | | | | | |
|  | # of DAPI-positive cells | | | | | | | | | | | | | |
|  | Un | | | | | | | | Tr | | | | | |
| IgG  (n = 4 rats) | 1462 ± 27.20 | | | | | | | | 1432 ± 84.10 | | | | | |
| Anti-CIM6P/IGF2R  (n = 4 rats) | 1516 ± 59.37 | | | | | | | | 1436 ± 21.29 | | | | | |
| Two-way RM ANOVA followed by Tukey's post-hoc test  Treatment: F (1, 12) = 0.2853, P = 0.6030  Training: F (1, 12) = 1.026, P = 0.3310  Treatment x Training interaction: F (1, 12) = 0.2120, P = 0.6534 | | | | | | | | | | | | | | |
|  | **Effect of training and anti-CIM6P/IGF2R on # of analyzed cells (c-Fos) DG** | | | | | | | | | | | | | |
|  | # of DAPI-positive cells | | | | | | | | | | | | | |
|  | Un | | | | | | | | Tr | | | | | |
| IgG  (n = 4 rats) | 2618 ± 94.73 | | | | | | | | 2554 ± 119.48 | | | | | |
| Anti-CIM6P/IGF2R  (n = 4 rats) | 2672 ± 126.28 | | | | | | | | 2608 ± 46.76 | | | | | |
| Two-way RM ANOVA followed by Tukey's post-hoc test  Treatment: F (1, 12) = 0.2818, P = 0.6052  Training: F (1, 12) = 0.3959, P = 0.5410  Treatment x Training interaction: F (1, 12) = 0.0, P > 0.9999 | | | | | | | | | | | | | | |
| **Fig. 6** | **Effect of training and anti-CIM6P/IGF2R on puromycin incorporation CA1** | | | | | | | | | | | | | |
|  | Puromycin levels (%) | | | | | | | | | | | | | |
|  | Un | | | | | | | | Tr | | | | | |
| IgG  (n = 4 rats) | 100 ± 3.33 | | | | | | | | 214.04 ± 29.45 | | | | | |
| Anti-CIM6P/IGF2R  (n = 4 rats) | 105.88 ± 8.59 | | | | | | | | 125.23 ± 6.77 | | | | | |
| Treatment: F (1, 12) = 6.891, P = 0.0222  Training: F (1, 12) = 17.83 ,P = 0.0012  Treatment x Training interaction: F (1, 12) = 8.983 ,P = 0.0111 | | | | | | | | | | | | | | |
|  | **Effect of training and anti-CIM6P/IGF2R on puromycin incorporation DG** | | | | | | | | | | | | | |
|  | Puromycin levels (%) | | | | | | | | | | | | | |
|  | Un | | | | | | | | Tr | | | | | |
| IgG  (n = 4 rats) | 100 ± 4.16 | | | | | | | | 208.19 ± 22.04 | | | | | |
| Anti-CIM6P/IGF2R  (n = 4 rats) | 98.80 ± 6.48 | | | | | | | | 115.97 ± 4.05 | | | | | |
| Two-way RM ANOVA followed by Tukey's post-hoc test  Treatment: F (1, 12) = 15.55, P = 0.0020  Training: F (1, 12) = 27.99, P = 0.0002  Treatment x Training interaction: F (1, 12) = 14.76, P = 0.0023 | | | | | | | | | | | | | | |
| **Fig. 7a** | **Effect of various M6P doses on IA memory** | | | | | | | | | | | | | |
|  | Mean Latency (s) | | | | | | | | | | | | | |
|  | T1 | | | | | | | | T2 | | | | | |
| Vehicle  (n = 12 rats) | 288.42 ± 32.53 | | | | | | | | 338.72 ± 39.97 | | | | | |
| 5 uM  (n = 7 rats) | 277.34 ± 40.90 | | | | | | | | 400.54 ± 48.80 | | | | | |
| 5 mM  (n = 12 rats) | 688.82 ± 49.27 | | | | | | | | 722.62 ± 48.36 | | | | | |
| 25 mM  (n= 6 rats) | 799.91 ± 67.98 | | | | | | | | 804.52 ± 71.13 | | | | | |
| 150 mM  (n = 7 rats) | 155.29 ± 39.17 | | | | | | | | 165.74 ± 24.07 | | | | | |
| Two-way RM ANOVA followed by Sidak's post-hoc test  Treatment: F (5, 49) = 21.84, P < 0.0001  Time: F (1, 49) = 0.9470 ,P = 0.3353  Treatment x Test interaction: F (5, 49) = 0.7209 ,P = 0.6109 | | | | | | | | | | | | | | |
| **Fig. 7b** | **Effect of Cre-injection and CIM6P/IGF2R ligands on CFC memory** | | | | | | | | | | | | | |
|  | Freezing (%) | | | | | | | | | | | | | |
|  | T1 | | | | | | | | T2 | | | | | |
| GFP/Veh  (n = 6 mice) | 41.67 ± 2.38 | | | | | | | | 50 ± 4.06 | | | | | |
| GFP/IGF2  (n = 6 mice) | 69.44 ± 3.44 | | | | | | | | 74.07 ± 2.75 | | | | | |
| GFP/M6P  (n = 5 mice) | 70.00 ± 2.22 | | | | | | | | 71.11 ± 2.08 | | | | | |
| Cre/Veh  (n= 7 mice) | 22.22 ± 1.71 | | | | | | | | 18.25 ± 1.59 | | | | | |
| Cre/IGF2  (n = 7 mice) | 22.22 ± 1.72 | | | | | | | | 15.87 ± 2.55 | | | | | |
| Cre/M6P  (n = 6 mice) | 22.2 ± 2.87 | | | | | | | | 15.74 ± 2.23 | | | | | |
| Two-way RM ANOVA followed by Sidak's post-hoc test  Treatment: F (5, 31) = 124.7 ,P < 0.0001  Time: F (1, 31) = 0.2209, P = 0.6417  Treatment x Test interaction: F (5, 31) = 6.891, P = 0.0002 | | | | | | | | | | | | | | |
